# Amyloid Beta-Mediated Epigenetic Alteration of Insulin-Like Growth Factor Binding Protein 3 Controls Cell Survival in Alzheimer's Disease

**DOI:** 10.1371/journal.pone.0099047

**Published:** 2014-06-25

**Authors:** Hye Youn Sung, Eun Nam Choi, Dahyun Lyu, Inhee Mook-Jung, Jung-Hyuck Ahn

**Affiliations:** 1 Department of Biochemistry, School of Medicine, Ewha Womans University, Seoul, Republic of Korea; 2 Tissue Injury Defense Research Center, School of Medicine, Ewha Womans University, Seoul, Republic of Korea; 3 Department of Biochemistry and Biomedical Sciences, Seoul National University College of Medicine, Seoul, Republic of Korea; University of S. Florida College of Medicine, United States of America

## Abstract

Swedish double mutation (KM670/671NL) of amyloid precursor protein (APP) is reported to increase toxic amyloid β (Aβ) production via aberrant cleavage at the β-secretase site and thereby cause early-onset Alzheimer's disease (AD). However, the underlying molecular mechanisms leading to AD pathogenesis remains largely unknown. Previously, our transcriptome sequence analyses revealed global expressional modifications of over 600 genes in APP-Swedish mutant-expressing H4 (H4-sw) cells compared to wild type H4 cells. Insulin-like growth factor binding protein 3 (*IGFBP3*) is one gene that showed significantly decreased mRNA expression in H4-sw cells. In this study, we investigated the functional role of *IGFBP3* in AD pathogenesis and elucidated the mechanisms regulating its expression. We observed decreased *IGFBP3* expression in the H4-sw cell line as well as the hippocampus of AD model transgenic mice. Treatment with exogenous IGFBP3 protein inhibited Aβ_1**–**42_- induced cell death and caspase-3 activity, whereas siRNA-mediated suppression of IGFBP3 expression induced cell death and caspase-3 cleavage. In primary hippocampal neurons, administration of IGFBP3 protein blocked apoptotic cell death due to Aβ_1**–**42_ toxicity. These data implicate a protective role for IGFBP3 against Aβ_1**–**42_-mediated apoptosis. Next, we investigated the regulatory mechanisms of IGFBP3 expression in AD pathogenesis. We observed abnormal *IGFBP3* hypermethylation within the promoter CpG island in H4-sw cells. Treatment with the DNA methyltransferase inhibitor 5-aza-2′-deoxycytidine restored *IGFBP3* expression at both the mRNA and protein levels. Chronic exposure to Aβ_1**–**42_ induced *IGFBP3* hypermethylation at CpGs, particularly at loci −164 and −173, and subsequently suppressed *IGFBP3* expression. Therefore, we demonstrate that expression of anti-apoptotic *IGFBP3* is regulated by epigenetic DNA methylation, suggesting a mechanism that contributes to AD pathogenesis.

## Introduction

Alzheimer's disease (AD) is the most common form of age-dependent dementia and is characterized by increased beta amyloid (Aβ) levels, extracellular senile plaques, intracellular neurofibrillary tangles, and massive neuronal loss in the brain. Senile plaques are deposits of Aβ that arise from abnormal sequential cleavage of amyloid precursor protein (APP) by β- and γ-secretases[1], [2]. Several genetic mutations have been reported in genes such as *APP*, presenilin 1 (*PSEN1*), and presenilin 2 (*PSEN2*) [1]. All of these mutations are associated with increased production of toxic Aβ and its secretion [2], [3] suggesting APP processing is the key causative factor in AD pathogenesis.

The Swedish mutation of *APP* harboring double mutations at codons 595 and 596 (APP_695_ numbering) substituting Lys-Met to Asn-Leu is a predominant form of familial Alzheimer's disease. Cells expressing Swedish mutant *APP* have dramatic increases (up to 6- to 8-fold) in Aβ_1**–**40_ and Aβ_1**–**42_ production via elevated endoproteolytic cleavage at the β-secretase site [4], [5]. Our previous study also found global alterations in gene expression, including genes associated with AD pathogenesis in *APP*-Swedish mutant cells [6]. Insulin-like growth factor binding protein-3 (*IGFBP3*) was one gene whose expression was significantly down-regulated in APP-Swedish mutant cells.

IGFBP3 is one of six IGFBP family members that bind peptide growth factors, such as insulin-like growth factor (IGF) -I and –II, with high affinity and thus regulates their biological actions, including cellular proliferation, differentiation, and metabolic activity by blocking subsequent IGF-I receptor activation [7], [8]. IGFBP3 has been widely studied because of its induction of apoptosis and/or suppression of cell proliferation. IGFBP3 attenuates the interaction between IGF-I and the IGF-I receptor, as well as via IGF-independent effectors such as the transforming growth factor (TGF)-βV receptor, tumor necrosis factor (TNF)-α receptor, retinoid X receptor (RXR)-α, and nuclear factor-κB (NF-κB) cascades [9], [10]. In contrast to the pro-apoptotic effects of IGFBP3, a number of studies have demonstrated that IGFBP3 stimulates cell proliferation and protects cells from apoptotic insults in a variety of cell types through IGF-dependent or -independent [11]–[14]. Furthermore, several studies have reported that IGFBP3 may facilitate cell survival or death in the same cellular system depending on the stimulus. IGFBP3 can be regulated by both TNF-α and IGF-I via different pathways in response to specific stimuli to regulate cell fate in bovine secretory mammary epithelial cells [15]. Another study also reported dual effects of IGFBP3 on survival and apoptosis in human umbilical vein endothelial cells. IGFBP3 enhanced doxorubicin-induced apoptosis but also promoted cell survival in serum-deprived conditions by regulating ceramide levels in these cells [16]. Accumulating evidence indicates that IGFBP3 is a multi-functional protein whose functions depend on the specific condition of the cell. However, the underlying molecular mechanisms of IGFBP3 biological action remain largely unknown.

IGFBP3 expression can be induced or suppressed by a variety of agents and factors, such as vitamin D, estrogen, androgen, retinoic acid, TGF-β, TNF-α, hypoxia, p53, and DNA methylation [9]. Several cancer studies have described aberrant hypermethylation in CpG islands of the I*GFBP3* promoter as one of the mechanisms responsible for *IGFBP3* gene silencing [17]–[19]. However, methylation-dependent epigenetic regulation of *IGFBP3* has not previously been investigated in AD.

In this study, we investigated the functional role of IGFBP3 using an AD model cell line. Furthermore, we elucidated the mechanism that regulates IGFBP3 expression and contributes to AD pathogenesis.

## Materials and Methods

### Cell culture

Human glioblastoma H4 cells and APP695-Swedish mutant (K595N/M596L)-expressing H4 cells (H4-sw) were kindly provided by Sangmee Ahn Jo's lab (Dankook University, Chungnam, Korea)and have been reported previously [20], [21]. H4 and H4-sw cells were cultured as previously described in Dulbecco's modified Eagle media (DMEM; Gibco/BRL) containing 10% fetal bovine serum (FBS; Gibco/BRL), 100 U/mL penicillin (Gibco/BRL), 100 µg/mL streptomycin (Gibco/BRL), and 2 mM L-glutamine (Gibco/BRL) [6]. To maintain H4-sw cells, 500 µg/mL geneticin (Gibco/BRL) was added to the growth media.

### Rat hippocampal neuronal culture

Hippocampal neuronal cultures were prepared from 3- to 6-day-old Sprague-Dawley rats. All procedures used in this study for handling and sacrificing the animals were in strict compliance with the guidelines of Korean animal protection law and approved by the Institutional Animal Care and Use Committee of Ewha Womans University School of Medicine (Permit Number: 13-0220). Briefly, hippocampi were dissected from 3- to 6-day-old rats into DMEM (Biowest), trypsinized for 1 hour at 37°C with 0.25% Trypsin/0.53 mM EDTA (Gibco/BRL), triturated with fire-polished Pasteur pipettes, and plated in 6-well plates coated with poly-L-lysine (Sigma-Aldrich) at a density of 4×10^5^ cells/well in Neurobasal media (Gibco/BRL) with 10% FBS. After 16 h, the media were changed to Neurobasal media supplemented with serum-free supplements containing 0.5 mM L-glutamine, 2% B-27, 1% N-2, and 1% penicillin/streptomycin (Invitrogen) for further culturing. Half of the media were changed every 2 days by aspirating and replacing it with fresh culture media for 14 days, at which time Aβ_1**–**42_ treatment was initiated.

### Mice

The APP swe/PS1 transgenic (Tg) mice (B6C3-Tg(APP695)85Dbo Tg(PSEN1)85Dbo) were originally purchased from The Jackson Laboratory and subsequently bred in the animal care facility at the College of Medicine, Ewha Womans University. These mice doubly express human APP carrying Swedish familial AD-linked mutations (K670N/M671L) and human Presenilin1 encoding a mutant exon 9-deleted variant (PSEN1/dE9). Twelve-month-old age-matched transgenic and wild-type (WT) littermates were used in experiments. They were sacrificed by cervical dicapitation under anaesthesia according to the Institutional Animal Care and Use Committee of Ewha Womans University School of Medicine approved procedures (Permit Number: 12-0206). Their brains were harvested and the frontal cortex, hippocampus, and cerebellum were isolated and freshly used for gene expression analyses.

### RNA extraction, reverse-transcription polymerase chain reaction (RT-PCR), and quantitative polymerase chain reaction (qPCR)

Total RNA was extracted using the RNeasy Plus Mini Kit (Qiagen) and RNeasy Lipid Tissue Mini Kit (Qiagen) for cultured cells and brain tissues, respectively, according to the manufacturer's protocol. Total RNA (1 µg) was converted to cDNA using Superscript II reverse transcriptase (Invitrogen) and oligo-(dT)_12**–**18_ primers (Invitrogen) according to the manufacturer's instructions. qPCR was performed in a 20-µl reaction mixture containing 1 µl cDNA, 10 µl SYBR Premix EX Taq (Takara Bio), 0.4 µl Rox reference dye (50×, Takara Bio), and 200 nM primers for each gene. The primer sequences were as follows: human *IGFBP3* (forward), 5′-CTCTGCGTCAACGCTAGTGC-3′; human *IGFBP3* (reverse), 5′-CGGTCTTCCTCCGACTCACT-3′; mouse *IGFBP3* (forward), 5′-CGAGTCTAAGCGGGAGACAG-3′; mouse *IGFBP3* (reverse), 5′-ACTTGTCCACACACCAGCAG-3′; human *GAPDH* (forward), 5′-AATCCCATCACCATCTTCCA-3′; human *GAPDH* (reverse), 5′-TGGACTCCACGACGTACTCA-3′ mouse *GAPDH* (forward), 5′-AATGTGTCCGTCGTGGATCT-3′; mouse *GAPDH* (reverse), 5′-GGTCCTCAGTGTAGCCCAAG-3′. Reactions were run on an ABI PRISM 7000 sequence detection system (Applied BioSystems) at 50°C for 2 min and 95°C for 10 min, followed by 40 cycles of 95°C for 15 sec and 60°C for 1 min, and a dissociation stage of 1 cycle at 95°C for 15 sec, 60°C for 20 sec, and 95°C for 15 sec. All PCR reactions were performed in triplicate, and the specificity of the reaction was detected by melting curve analyses at the dissociation stage. Comparative quantification of each target gene was performed based on the cycle threshold (C_T_), which was normalized to *GAPDH* using the ΔΔC_T_ method.

### Western blot analyses

Proteins (40–50 µg) were resolved using denaturing 10 or 15% sodium dodecyl sulfate- polyacrylamide gel electrophoresis (SDS-PAGE) and transferred to polyvinylidene fluoride (PVDF) membranes. Membranes were blocked in 5% skim milk in Tris-buffered saline with 0.1% Tween 20 (TBST) and subsequently incubated overnight at 4°C with the following primary antibodies: goat anti-IGFBP3 polyclonal antibody (1∶2000, R&D Systems), rabbit anti-cleaved caspase 3 polyclonal antibody (1∶1000, Cell Signaling), and mouse anti-β-actin monoclonal antibody (1∶2000, Santa Cruz). After washing, the membranes were incubated with secondary antibodies conjugated to horseradish peroxidase for 1 h at room temperature. Chemiluminescence was detected using Super Signal West Dura substrate (Thermo Scientific) according to the manufacturer's protocol. Bands were visualized using a Luminescent Image analyser LAS-300 (General Electric) and quantified using Image Gauge software (Science Lab)

### Small interfering RNA (siRNA) transfection

Pre-designed siRNA for *IGFBP3* (siBP3, CAT#ID L-004777-00-0005) and a non-targeting control (siNC, CAT#ID D-001206-13-05) were purchased from Thermo Scientific. To deplete *IGFBP3* expression, H4 cells were either transfected with 100 nM siBP3 or siNC using the DharmaFECT 4 transfection reagent (Thermo Scientific) according to the manufacturer's protocol. Knockdown of *IGFBP3* expression was confirmed using qPCR 24 h post-transfection and western blot analyses 48 h post-transfection.

### Flow cytometric apoptosis analyses

Apoptosis-induced cells or untreated cells were trypsinized with 0.25% trypsin/0.53 mM EDTA and harvested by centrifugation (200 × g, 5 min, 4°C). After two washes in phosphate-buffered saline (PBS) containing 0.1% glucose, cells were resuspended in propidium iodide (PI) staining buffer (0.1% sodium citrate, 0.1% Triton X-100, 10 µg/ml PI, and 10 µg/ml RNase A) and incubated for 15 min in the dark. Cell-cycle profiles were determined counting 20,000 cells using a FACSCalibur (Becton Dickinson). The percentage of apoptotic cells was analyzed using Modfit LT software (Verity Software House) as the percentage of cells in the sub-G1 phase.

### Bisulfite conversion

Bisulfite modified gDNA was prepared using the EpiTech Bisulfite kit (Qiagen, catalog #59104) according to the manufacturer's instructions. The bisulfate reaction was performed on 2.0 µg gDNA and the reaction volume was adjusted to 20 µl with sterile water and 120 µl of conversion reagent, containing 85 µl of Bisulfite mix and 35 µl of DNA protect buffer, was added. The sample tubes were placed in a thermal cycler (MJ Research) and the following steps were performed: 5 min at 95°C, 25 min at 60°C, 5 min at 95°C, 85 min at 60°C, 5 min at 95°C, 175 min at 60°C and stored at 20°C. The converted samples were mixed with 560 µl of the freshly prepared Buffer BL and added into the column. The column was centrifuged at full speed for 1 min and discarded the flow-through. The column was then washed by adding 500 µl of Buffer BW and incubated with 500 µl of Buffer BD at room temperature (15∼25°C) for 15 min. After incubation, the column was centrifuged at full speed for 1 min. The column was again washed by adding 500 µl of Buffer BW and spun at full speed (this step was repeated). The converted gDNA was eluted by adding 20 µl of Buffer EB into the column and spin. The bisulfite converted gDNA samples were stored at −20°C until methylation analysis.

### Bisulfite sequencing analyses of DNA methylation patterns using the 454 GS-FLX system

Bisulfite PCR of the *IGFBP3* promoter regions was performed in 50 µl reactions containing 10 ng bisulfite-modified genomic DNA, 1.5 mM MgCl_2_, 200 µM dNTPs, 1 U Platinum Taq polymerase (Invitrogen), 1× Platinum Taq buffer, and 200 nM each specific BSP forward and reverse primers. The BSP primers were designed using MethPrimer software. The bisulfite *IGFBP3* PCR product was 350 bp (position in the human GRCh37/hg19 assembly: ch7 45,960,812–45,961,161) and contained 38 cytosine-phosphate-guanine (CpG) sites. Sequences of the bisulfite PCR primers are: 5′-GTTGTGGAATTTAGGTAGGAAG-3′ (forward) and 5′-AAAATACTAAAATAACCTAAAATACC-3′ (reverse). The reaction was run at 95°C for 5 min, followed by 30 cycles at 95°C for 30 sec, 55°C for 30 sec, and 72°C for 30 sec. There was a final elongation step at 72°C for 5 min. Bisulfite PCR products were purified using QIAquick Gel Extraction kits (Qiagen) according to the manufacturer's protocol.

A library was prepared according to the GS FLX titanium library prep guide using bisulfite PCR products. Libraries were quantified using Ribogreen assays (Invitrogen). The emPCR, corresponding to clonal amplification of the purified library, was performed using the GSFLX titanium emPCR Kit (Roche/454 Life Sciences). Briefly, libraries were immobilized onto DNA capture beads. The library-beads obtained were added to a mixture of amplification mix and oil, and vigorously shaken on a Tissue LyserII (Qiagen) to create "micro-reactors" containing both amplification mix and a single bead. The emulsion was dispensed into 96-well plates and the PCR amplification program was run according to the manufacturer's recommendations. Following amplification, the emulsion was chemically broken and beads carrying the amplified DNA library were recovered and washed by filtration. Positive beads were purified using the biotinylated primer A (complementary to adaptor A), which binds streptavidin-coated magnetic beads. DNA library beads were then separated from magnetic beads by melting the double-stranded amplification products, leaving a population of bead-bound single-stranded template DNA fragments. The sequencing primer was then annealed to the amplified single-stranded DNA. Finally, beads carrying amplified single-stranded DNA were counted using a Particle Counter (Beckman Coulter). Sequencing was performed on a Genome Sequencer FLX titanium (Roche/454 Life Sciences), and each sample was loaded in one region of a 70 mm×75 mm Pico Titer plate (Roche/454 Life Sciences) fitted with an 8-lane gasket.

For data analyses, we used Amplicon Variant Analyzer (AVA) software (Roche). The report includes auto-detected variants using the "computation load detected variants" command from the command line interface (CLI). The auto-detected variants show the frequency at which all the variants defined in the project were observed.

### Pyrosequencing for DNA methylation analyses

We used bisulfite pyrosequencing for methylation analyses of the *IGFBP3* target region. Each primer was designed using the PSQ assay design program (Qiagen, USA). Primer sequences were as follows: 5′-AGAAGTAGGGGTGGTTTAGGAT-3′ (forward), 5′- AAATAACCCAACACACCTTAATTCTTATAA-3′ (biotinylated-reverse), 5′- GTTAGTGTTTAGTTTTGAGTAG-3′ (sequencing primer). PCR reactions were performed in a volume of 20 µl with 20 ng or less bisulfite-converted gDNA, 10 µl 2× Hot/Start PCR premix (Enzynomics), 1 µl forward primer (10 pmole/µl), and 1 µl biotinylated-reverse primer (10 pmole/µl). Amplifications were performed according to the general guidelines suggested by pyrosequencing: denaturating at 95°C for 10 min, followed by 50 cycles at 95°C for 30 sec, 56°C for 30 sec, 72°C for 30 sec, and a final extension of 72°C for 10 min. The PCR reaction (2 µl) was confirmed by electrophoresis in a 2.5% agarose gel and visualized by ethidium bromide staining.

ssDNA template was prepared from 16–18 µl of biotinylated PCR product using streptavidin Sepharose HP beads (Amersham Biosciences), following the PSQ 96 sample preparation guide using multichannel pipets. The respective sequencing primers (15 pM) were added for analyses. Sequencing was performed on a PyroMark ID system using the Pyro Gold reagent kit (Biotage) according to the manufacturer's instructions. The sequence to analyze was TYGGGGTYGAGTTYGGGGGYGTGTAGTTYG (position in the human GRCh37/hg19 assembly: ch7 45,961,016–45,961,045). The methylation percentage was calculated by the average degree of methylation at five CpG sites formulated in pyrosequencing.

### Bisulfite sequencing analyses

Bisulfite PCR products of the *IGFBP3* promoter regions (position in the human GRCh37/hg19 assembly: ch7 45,960,812–45,961,161) were purified using QIAquick Gel Extraction kits (Qiagen) according to the manufacturer's protocol and ligated into the yT&A cloning vector (Yeastern Biotech). The ligation products were used to transform competent DH5α *Esherichia coli* cells (RBC Bioscience) using standard procedures. PCR product-positive clones were confirmed by colony PCR using bisulfite PCR primers to verify insert size. Plasmid DNA was then extracted from at least twenty insert-positive clones using QIAprep Spin Miniprep kits (Qiagen) and sequenced using M13 primers to analyze the methylation status at specific CpG sites.

### 5-aza-2′-deoxycytidine (5-aza-dC) treatment

To demethylate methylated CpG sites, H4-sw cells were treated with 10 µM 5-aza-2′-deoxycytidine (Sigma-Aldrich) for 3 days. The media was replaced daily.

### Amyloid-β (Aβ_1–42_) preparation and treatment

Synthetic Aβ_1**–**42_ (American Peptide) was dissolved in hexafluoro-2-propanol (HFIP, Sigma-Aldrich) for 72 h in the dark and aliquoted into microcentrifuge tubes. HFIP was removed by evaporation in a vacuum concentrator (Hanil). The tubes were stored at −80°C until use. To prepare a soluble form of Aβ_1**–**42_, dried Aβ_1**–**42_ was dissolved in dimethylsulfoxide (DMSO, Sigma-Aldrich) to make a 1 mM stock solution. The Aβ_1**–**42_ stock solution was immediately diluted in serum-free media to make final 10 nM to 5 µM working solutions. H4 cells were treated with soluble Aβ_1**–**42_ at different concentrations for various time points. To prepare the oligomeric form of Aβ_1**–**42_, the soluble stock solution was first diluted in phosphate-buffered saline (PBS) to a concentration of 10 µM, mixed vigorously, and incubated at 4°C for 24 h. Aβ_1**–**42_ peptides oligomerize to exhibit increased soluble oligomeric bands with higher molecular weight accompanied with decreased monomeric bands (Figure S1). The oligomeric form of Aβ_1**–**42_ was further diluted in supplements-free media to a final concentration of 500 nM. Rat hippocampal neuronal cells were treated with oligomeric Aβ_1**–**42_ at 500 nM for 24 h.

### Statistical analysis

All data are expressed as mean ± standard deviation of at least three independent experiments. Statistical analyses were carried out using GraphPad Prism5 software and the details of the statistical analysis for each data set were included in the figure legends. *P* values less than 0.05 were considered statistically significant.

## Results

### Altered *IGFBP3* expression in APP-Swedish mutant cells and APP swe/PSEN1 transgenic mice

**Figure 1:**
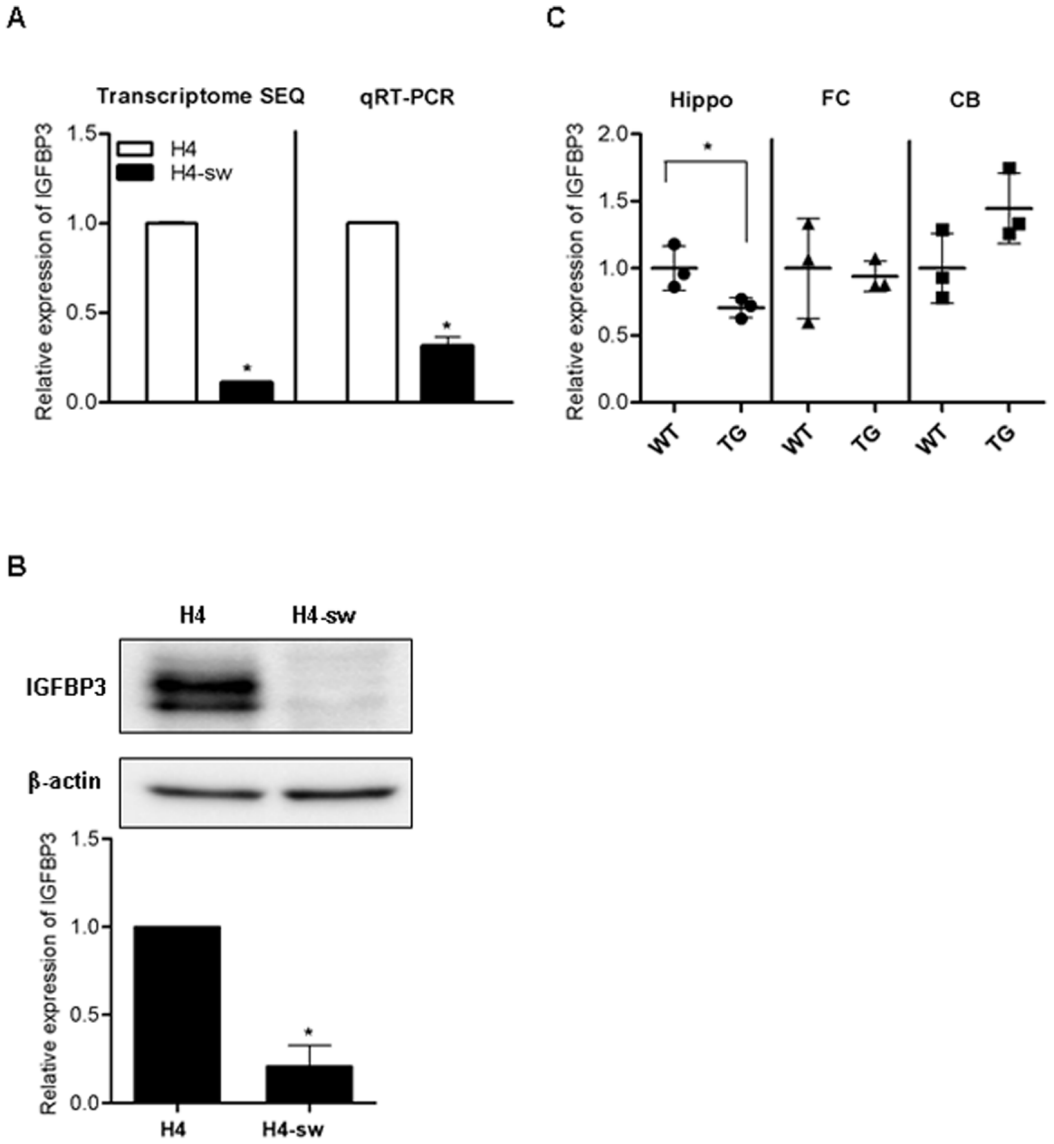
IGFBP3 expression is down-regulated in an APP-mutant cell line and transgenic mice *IGFBP3* mRNA expression in normal and APP-Swedish mutant H4 cells (A), and the brain of wild type and PSEN1-APP transgenic mice (B6C3-Tg(APP695)85Dbo Tg(PSEN1)85Dbo) (C) was measured by transcriptome sequencing analyses and qPCR. Expression of IGFBP3 protein in normal and APP-Swedish mutant H4 cells was detected using western blot analyses (B). IGFBP3 protein in APP-Swedish mutant H4 cells is expressed relative to IGFBP3 protein levels in normal H4 cells. Data are represented as the mean ± standard deviation (SD) of triplicate experiments. Statistical analyses were performed using *t*-tests (* indicates p<0.05). H4-sw, APP-Swedish mutant H4 cells; Hippo, hippocampus; FC, frontal cortex; CB, cerebellum.

The APP-Swedish mutant-harboring H4 (H4-sw) cells secrete 1.2–1.7 ng/mL of Aβ_1**–**40_ and 147–243 pg/mL of Aβ_1**–**42_ while 4.4–6.7 pg/mL of Aβ_1**–**40_ and 1.54–13.35 pg/mL of Aβ_1**–**42_ were detected in H4 cells (Figure S2). Previously, we found that the expression of over 600 genes was altered in H4-sw cells compared to wild type H4 cells using transcriptome sequencing analyses [6]. Among these genes, *IGFBP3* expression was down-regulated approximately 10-fold in H4-sw cells (Figure 1A). In agreement with our transcriptome sequencing data, *IGFBP3* mRNA levels (Figure 1A) as well as protein levels (Figure 1B) were approximately 3.5-fold and 5-fold decreased in H4-sw cells, respectively, compared to wild type H4 cells. *IGFBP3* mRNA expression levels were measured in the brains of APPswe/PSEN1 double mutant transgenic mice. Significantly decreased mRNA levels of *IGFBP3* were observed in hippocampus of the transgenic mice. However, *IGFBP3* mRNA levels remained unchanged in the frontal cortex and increased in cerebellum of the transgenic mice, but the increase was not statistically significant (Figure 1C).

### IGFBP3 protects cells from Aβ_1–42_-induced apoptosis

**Figure 2:**
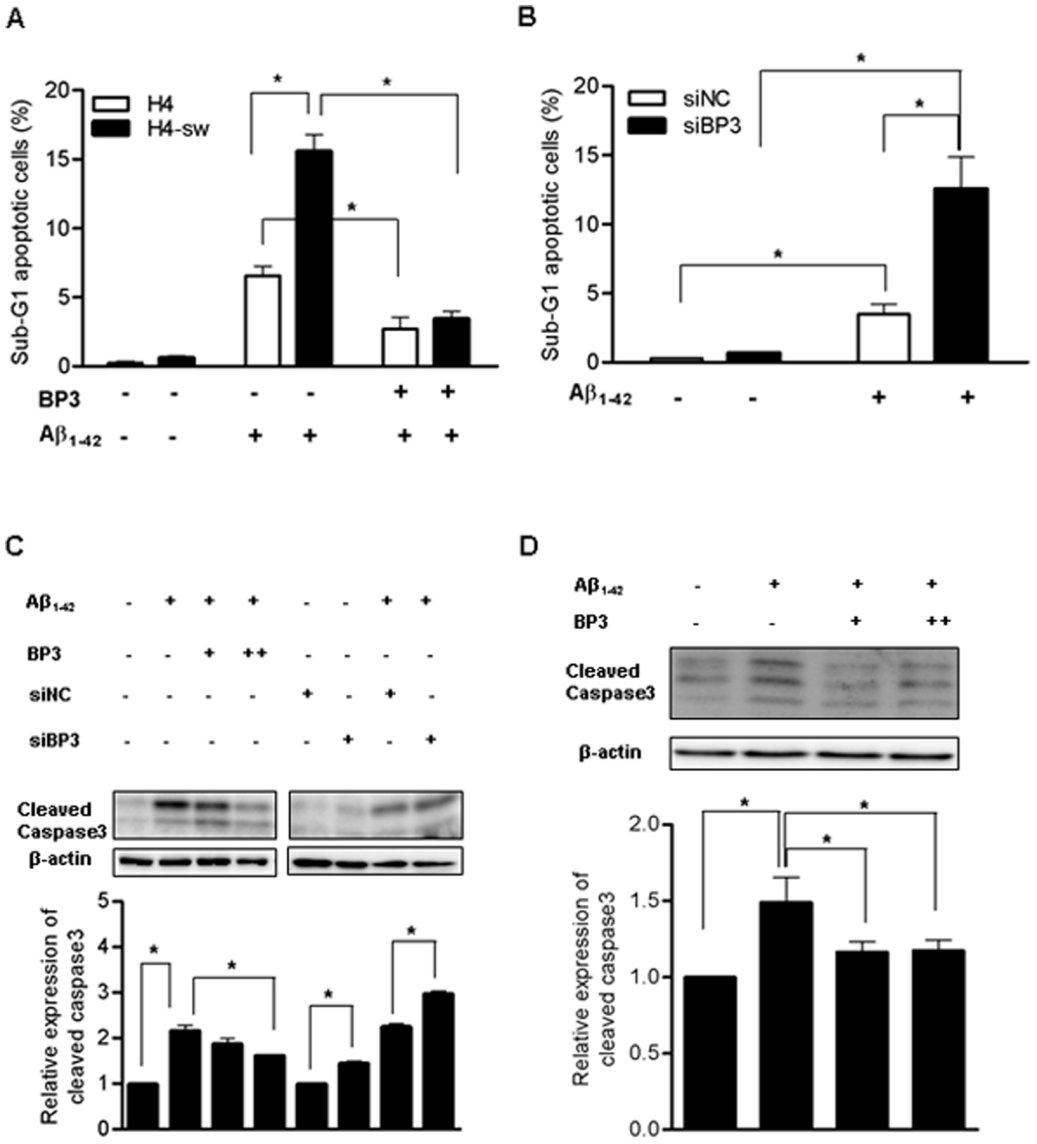
IGFBP3 protects cells from Aβ_1–42_ induced apoptosis H4 and H4-sw cells were treated for 24 h in serum-free media (SF) with 5 µM soluble Aβ_1__–42_ in the presence or absence of exogenous recombinant human IGFBP3 (BP3) protein. *IGFBP3* knockdown H4 cells generated by siRNA transfection or non-targeted siRNA-transfected H4 cells were treated with or without 5 µM soluble Aβ_1**–**42_ in SF media for 24 h. Apoptosis was evaluated after propidium iodide staining using FACS analyses. Compiled FACS results from three independent experiments are graphically illustrated in (A) and (B). Caspase 3 activation was examined in H4 cells by detecting cleaved forms of caspase 3 using western blot analyses. Representative results are illustrated and graphical values from densitometric analyses after normalization to β-actin are reported as relative values to that of untreated control (C). Rat hippocampal neuronal primary cells in supplements-free media were treated for 24 h with 500 nM oligomeric Aβ_1**–**42_ in the presence or absence of exogenous recombinant human IGFBP3 (BP3) protein. Caspase 3 activation was determined by detecting cleaved forms of caspase 3 using western blot analyses (D). Data are the mean ± SD of three independent experiments. Statistical analyses were performed using two-way analysis of variance (ANOVA) and Bonferroni post-tests. * indicates p<0.05. H4-sw, APP-Swedish mutant H4 cells; BP3, IGFBP3; siNC, non-targeting siRNA; siBP3, IGFBP3 siRNA.

It is widely known that IGFBP3 plays a pivotal role in both cell growth and apoptosis. Recent studies have shown that IGFBP3 either inhibits or promotes apoptotic cell death depending on the cell type and inducing agent [10], [16]. To investigate the role of IGFBP3 in the development of AD, we induced apoptosis in H4 and H4-sw cells using Aβ_1**–**42_ in the presence or absence of exogenous recombinant human IGFBP3. After 24 hours of Aβ_1**–**42_ treatment in the presence or absence of IGFBP3 in serum-free media, we measured cell viability. Our FACS results showed that the viability of H4-sw cells with down-regulated IGFBP3 expression decreased approximately 2.5-fold compared to H4 cells. Additionally, treatment with exogenous IGFBP3 (2 µg/ml) can inhibit Aβ_1**–**42_-induced cell death in both H4 and H4-sw cells (Figure 2A). Further experiments revealed that knocking down IGFBP3 in H4 cells using siRNA rendered the cells more susceptible to Aβ_1**–**42_- induced toxicity (Figure 2B). Consistent with our FACS results, caspase 3 cleavage in response to Aβ_1**–**42_-induced cell death was inhibited by adding IGFBP3 protein to wild type H4 cells, whereas caspase 3 activation was promoted in IGFBP3-knockdown H4 cells (Figure 2C). These findings indicate that IGFBP3 has a protective effect against Aβ_1**–**42_-induced apoptosis.

We also examined the effects of IGFBP3 on primary neuronal cells. We induced apoptosis in rat hippocampal neuronal cells by treating cells with 500 nM oligomeric Aβ_1**–**42_ for 24 h under supplements-free conditions. Addition of exogenous IGFBP3 (1 and 2 µg/ml) significantly protected neurons from Aβ_1**–**42_-induced apoptosis with a concomitant decrease in caspase 3 activation (Figure 2D). Endogenous *Igfbp3* mRNA and protein expressions have not been altered by oligomeric Aβ_1**–**42_ treatment for 24h (Figure S3).

### *IGFBP3* expression is regulated by DNA methylation

A number of studies have reported that epigenetic modifications such as DNA methylation and histone acetylation play important roles in *IGFBP3* gene silencing in several human cancers [10], [22]. Therefore, we examined the *IGFBP3* methylation status of CpG sites within its promoter region in H4 and H4-sw cells to investigate whether epigenetic modifications are major mechanisms that control *IGFBP3* expression.

**Figure 3:**
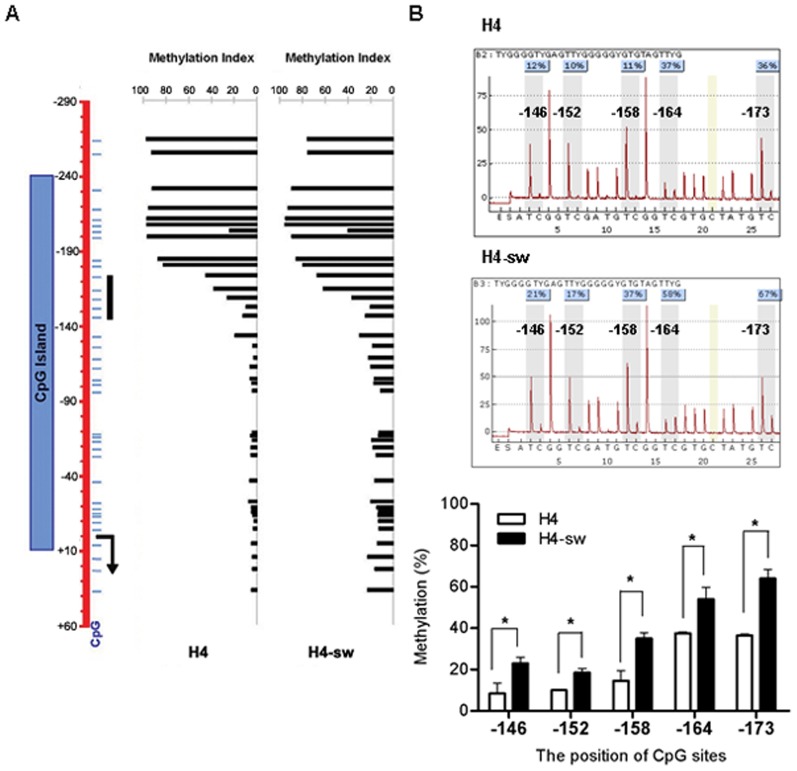
Hypermethylation of CpG islands within the *IGFBP3* promoter in APP-Swedish mutant cells Schematic diagram of the genomic region (+60 to −290) of IGFBP3 that was analyzed for methylation status. The CpG island is represented as a box (−240 to +10). Thin horizontal lines indicate each CpG site. The bent arrow indicates the transcription start site (+1) and the thick vertical solid line indicates the target CpG sites for pyrosequencing analyses (A). Methylation status analyses were conducted using bisulfite sequencing analyses, the 454 GS-FLX system, and bisulfite pyrosequencing. Individual bars represent the percentage of methylation at the corresponding CpG site within the *IGFBP3* promoter (A). Representative pyrograms are shown for each sample with the percentage methylation at each of the five CpG sites tested (B). Average percent methylation of triplicate pyrosequencing analyses at each of the five CpG sites are presented graphically (B). Data are shown as the mean ± SD of triplicate experiments. Statistical analyses were performed using *t*-tests (* indicates p<0.05). H4-sw, APP-Swedish mutant H4 cells.

**Figure 4:**
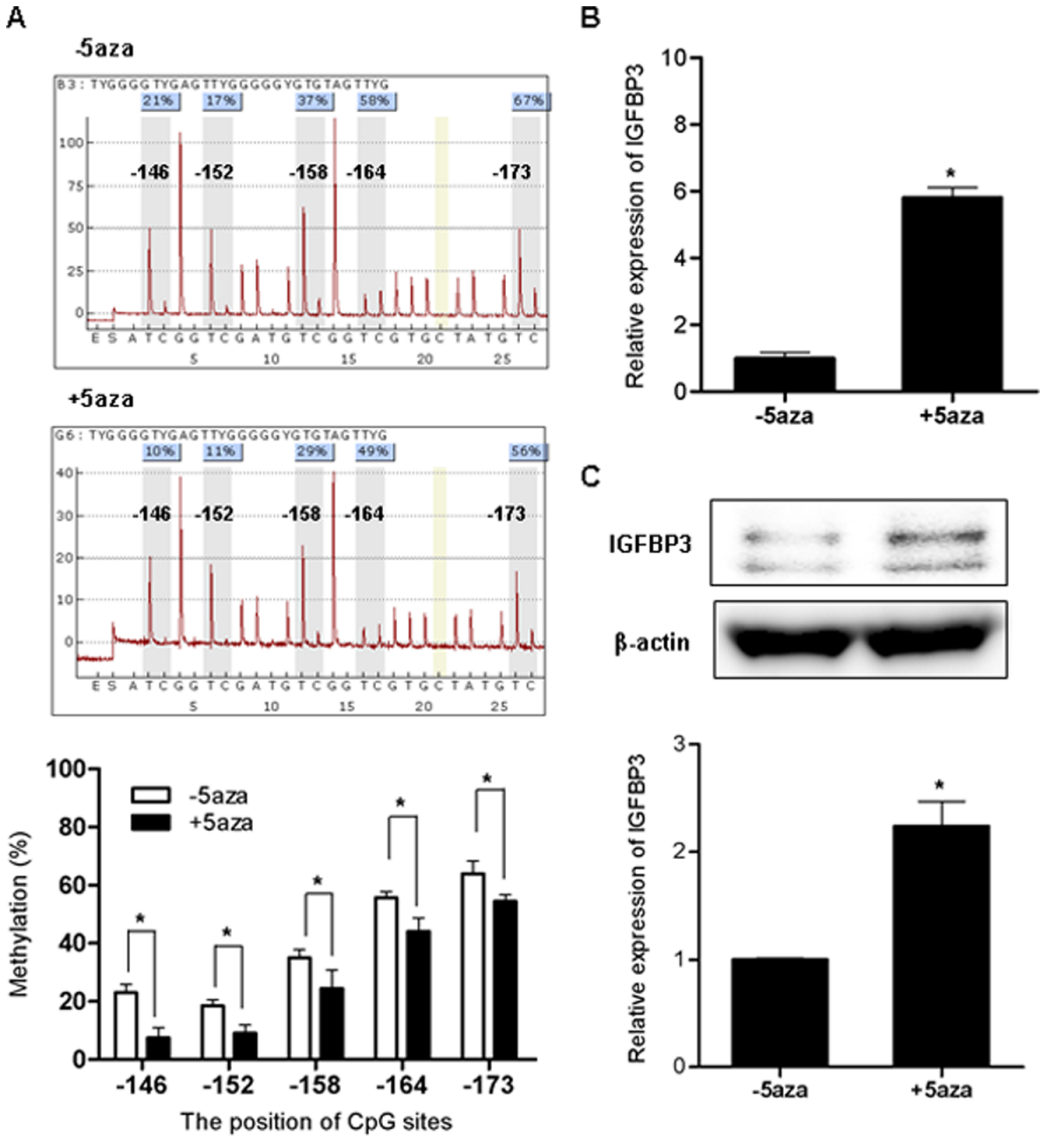
*IGFBP3* expression changes following demethylation in APP-Swedish mutant cells H4-sw cells were treated for 3 days with 10 µM 5-aza-2′-deoxycytidine. DNA methylation status at specific CpG sites were analyzed using bisulfite pyrosequence analyses. The average percent methylation of triplicate pyrosequencing analyses from each of the five CpG sites are presented graphically (A). After treatment with 5-aza-dC, *IGFBP3* mRNA expression was measured using qPCR (B). IGFBP3 protein expression was detected using western blot analyses. Representative results are illustrated and values from densitometric analyses after normalization to β-actin are reported relative to that of untreated controls (C). Data are shown as the mean ± SD (n = 3). Statistical analyses were performed using *t*-tests (* indicates p<0.05). 5aza, 5-aza-2′-deoxycytidine.

The *IGFBP3* promoter region (+60 to −290) was PCR amplified using bisulfide-modified genomic DNA as a template to obtain 350 bp bisulfite PCR products in H4 and H4-sw cells (Figure 3A). DNA methylation patterns of PCR amplicons were analyzed using the 454 GS-FLX system. Our results show that promoter CpGs (25 out of 32) within the CpG island were hypermethylated in H4-sw cells compared to H4 cells (Figure 3A). Further analyses of DNA methylation patterns using bisulfite pyrosequences also revealed higher DNA methylation at the five CpG sites tested (located in −146, −152, −158, −164, and −173 from the transcriptional start site) in H4-sw cells than in wild type H4 cells (Figure 3B). To determine whether transcriptional silencing of *IGFBP3* is regulated by DNA methylation of the promoter CpG island, we treated H4-sw cells with the DNA methyltransferase inhibitor 5-aza-2′-deoxycytidine. After treatment with 10 µM 5-aza-2′-deoxycytidine, decreased methylation activity of the H4-sw promoter was confirmed using pyrosequencing analyses (Figure 4A) and concomitantly, expression of *IGFBP3* mRNA and protein were dramatically upregulated in H4-sw cells (Figure 4B and 4C).

### Epigenetic modifications caused by Aβ_1–42_ alters *IGFBP3* expression

**Figure 5:**
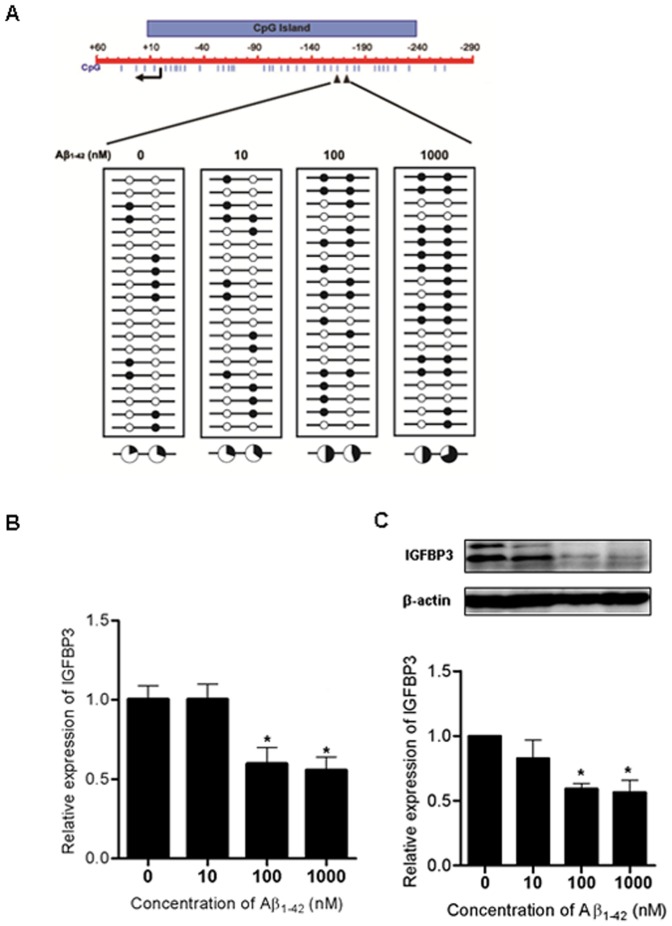
CpG island methylation is altered in the *IGFBP3* promoter region in H4 cells treated with Aβ_1–42_ H4 cells were treated for 5 days with different concentrations of Aβ_1__–42_. DNA methylation at the -164 and -173 CpG sites was analyzed using bisulfite sequencing analyses (A). Each circle represents CpG dinucleotides. The methylation status of each CpG site is illustrated by black (methylated) and white (unmethylated) circles. The total percentage of methylation at specific CpG sites is indicated as a pie graph. The black segment of the pie graph indicates methylated CpG percentage whereas the white segment represents the unmethylated CpG percentage (A). *IGFBP3* expression after treatment with Aβ_1**–**42_ was determined using qPCR (B) and western blot analyses (C). Graphs depict compiled data from three independent experiments and values are relative to those of untreated controls. Data are shown as the mean ± SD (n = 3). Statistical analyses were performed using one-way ANOVA and Bonferroni post-tests (* indicates p<0.05).

After treatment with various concentrations of Aβ_1**–**42_ (0, 10, 100, and 1000 nM) for 5 days, the DNA methylation status was determined using bisulfite sequencing analyses. DNA methylation of the *IGFBP3* promoter CpG sites, located −164 and −173 from the transcriptional start site, was increased at the 100 nM concentration of Aβ_1**–**42_ compared to untreated controls in H4 cells. The maximum increase was detected at 1000 nM Aβ_1**–**42_ (Figure 5A). *IGFBP3* mRNA and protein expression were significantly decreased when H4 cells were treated with greater than 100 nM Aβ_1**–**42_ (Figure 5B and 5C). These results indicate that Aβ_1**–**42_ treatment can induce aberrant hypermethylation in the *IGFBP3* promoter region and subsequently, suppress *IGFBP3* expression.

## Discussion

H4-sw cells were used in this study and are an AD model cell line that exhibits high levels of secreted toxic forms of Aβ, such as Aβ_1**–**40_ and Aβ_1**–**42_. Moreover, changes in the expression of genes associated with AD pathogenesis, including presenilin 2, AQP1, glycogen synthase kinase 3, and cyclin-dependent kinase 5, occur in these cells [6]. Previously, our transcriptome sequence analyses revealed that in addition to these AD-associated genes, expression of over 600 genes was altered in H4-sw cells compared to H4 cells [6]. *IGFBP3* was one of the genes whose expression was significantly altered in H4-sw cells. As shown in Figure 1, *IGFBP3* mRNA and protein expression were dramatically reduced in H4-sw cells (Figure 1A, 1B). Similarly, decreased *IGFBP3* expression was also detected in the brain, particularly the hippocampus, of APP swe/PSEN1 transgenic mice (Figure 1C). Numerous amyloid deposits in the hippocampus and the cortex have been previously described in these mice at 12 months of age [23]. Interestingly, a large scale clinical study has demonstrated a significant association between low IGFBP3 serum levels and cognitive impairment, independent of low IGF-I serum levels, in elderly male AD patients compared to patients with mild cognitive impairment (MCI) and normal controls [24]. In contrast to our results, Rensink *et al.* reported increased IGFBP3 expression in Dutch mutant Aβ_1**–**40_-treated human brain pericytes as well as in a subset of AD brain regions, particularly in senile plaques and cerebral amyloid angiopathy of the cortex but not of the hippocampus [25]. This discrepancy may be explained by the multi-functional properties of IGFBP3 that result in different biological actions depending on the cell type, experimental conditions, and inducing agents [9].

IGFBP3 was originally identified as a protein that binds with IGFs to inhibit their growth-stimulating and survival actions by sequestering them from the IGF receptor [9], [10]. Despite the well-established growth inhibitory and pro-apoptotic activities of IGFBP3, accumulating evidence indicates that IGFBP3 is also involved in cell growth and survival in many cell types via IGF-dependent or -independent mechanisms [11]–[14]. Furthermore, several studies have demonstrated the pleiotropic role of IGFBP3 in promoting and/or preventing cell survival to response to different insults in the same cellular system [15], [16]. The neuroprotective effects of IGF-1 have been reported in relation to AD. IGF-1 protects neuronal cells against Aβ-induced toxicity in an *in vitro* system [26] and decreases cognitive impairment and Aβ deposition in AD transgenic mice [27]. These protective roles of IGF-1 may be interrupted by forming complex with IGFBP3, leading to decreased IGF-1 bioavailability and transport into central nervous system [25], [28]. Because serum IGF-1 can transport into the brain via the blood-brain barrier, alteration in the serum levels of IGF-1 and/or IGFBPs may change IGF-1 input to the brain. Some clinical studies have shown lowered circulating IGF-1 levels in AD patients carrying the Swedish mutation [29] and late-onset AD group [30]. However, there are also discrepant results that observed higher total circulating IGF-1 levels and higher molar ratios of IGF-1/IGFBP3 (an indicator of bioavailable IGF-1) in the AD group compared to the age-matched healthy group [31], [32]. Recently, Duron et al. reported that low IGF-1 and IGFBP3 serum levels were significantly associated with cognitive status in men but not in women. There were no differences observed in bioactive IGF-1 levels between AD and MCI patients and controls in a large-size population [24]. The complicated consequences related to the IGF-1/IGFBP3 system and AD progression still remains to be elucidated.

Importantly, IGFBP3 can also be involved in neuronal survival and protection via IGF-1-independent mechanisms. IGFBP3 has been identified as a binding partner of humanin, a known survival peptide that protects neuronal cells against Aβ cytotoxicity and cell death. Humanin-IGFBP3 binding markedly potentiated the survival ability of humanin from Aβ_1**–**43_ toxicity in primary murine cortical neurons through an IGF-1-independent pathway [33]. In the present study, our results also indicate a protective role for Igfbp3 in primary rat hippocampal neurons (Figure 2D). Apoptosis induced by the oligomeric form of Aβ_1**–**42_ was significantly attenuated when cells were supplemented with recombinant IGFBP3. In contrast to a previous study showing a pleiotrophic nature depending on cell type [33], our results indicate an anti-apoptotic role for IGFBP3 in AD progression in primary rat neurons as well as neuroglioma cells. APP-Swedish mutant neuroglioma cells, which have IGFBP3 expression down-regulated compared to wild type H4 cells, were sensitive to Aβ_1**–**42_-induced cytotoxicity more than wild type H4 cells. The addition of exogenous human recombinant IGFBP3 significantly attenuated cell death and caspase 3 activity. Knockdown of endogenous *IGFBP3* using RNA interference promoted cell death and enhanced caspase 3 activity in H4 cells, indicating a protective effect of IGFBP3 in Aβ_1**–**42_-induced apoptosis (Figure 2).

Transcriptional regulation of IGFBP3 is mediated by alterations in the activity of transcription factors, such as p53, or by epigenetic modifications, such as DNA methylation and histone modification [10]. Silencing of *IGFBP3* expression through hypermethylation of CpG islands within the promoter region has been described in several cancers, including gastric, breast, colorectal [19] ovarian [34], and hepatocellular carcinomas [35]. However, epigenetic regulation of *IGFBP3* has not been reported in AD. Recent evidence supports an association between aberrant DNA methylation and AD development. Increased levels of S-adenosylhomocysteine, a methyltransferase inhibitor, have been detected in AD brains compared to age-matched controls, and are closely related to the cognitive impairment of patients [36]. Hypomethylation-dependent overexpression of several genes involved in the biogenesis and accumulation of amyloid plaques, such as *APP*, *PSEN1*, and β-secretase (*BACE*), has also been reported in AD brains [37], [38]. Therefore, we investigated whether epigenetic regulation of *IGFBP3* would be one mechanism causing aberrant expression during AD progression. Our results clearly show hypermethylation of CpG islands within the *IGFBP3* promoter region (+60 to −290) in H4-sw cells compared to wild type H4 cells (Figure 3). Treatment with the DNA methyltransferase inhibitor 5-aza-2′-deoxycytidine restored suppressed *IGFBP3* expression, strongly supporting DNA methylation-dependent transcriptional regulation of *IGFBP3* (Figure 4).

In cancer studies, it has been shown that IGFBP3 functions as a tumor suppressor, and hypermethylation-mediated *IGFBP3* gene silencing promotes cancer progression. *IGFBP3* hypermethylation is significantly associated with poor prognosis in stage I non-small-cell lung cancer [39]. Patients with low *IGFBP3* expression and high *IGFBP3* promoter methylation have strong correlations with low survival rates in ovarian endometrioid carcinoma, an ovarian cancer subtype [40]. This study also revealed an association between p53 and *IGFBP3* promoter methylation by identifying four critical hypermethylated p53 binding sequences in the promoter (−210, −206, −183, and −179) that are essential for p53-dependent *IGFBP3* transcriptional activity [40].

A previous study demonstrated that Aβ can induce aberrant epigenetic alterations, such as global hypomethylation and hypermethylation of certain genes. For example, neprilysin (*NEP*), an enzyme responsible for clearance of Aβ accumulation, is aberrantly altered in cerebral endothelial cell cultures [41]. It has been suggested that Aβ-induced aberrant epigenetic modifications may contribute to further Aβ accumulation and cell damage via overexpression of AD-associated genes such as *APP*, *PSEN1*, *PSEN2*, and *BACE*, as well as suppressed expression of Aβ degrading enzyme genes, thereby resulting in a vicious cycle [41]. Our results have also proven abnormal epigenetic alterations mediated by toxic Aβ. As shown in Figure 5, promoter CpG methylation was significantly increased, particularly at the CpGs located −164 and −173 from the transcriptional start site in H4 cells treated long term with low Aβ_1**–**42_ concentrations. However, treatment with high concentrations (greater than 1 µM) for 1 or 2 days did not affect *IGFBP3* promoter DNA methylation (data not shown). The altered DNA methylation status at the two CpG sites upon Aβ_1**–**42_ treatment correlated with suppressed *IGFBP3* mRNA and protein expression (Figure 5). This result suggests that even low concentrations of Aβ may induce abnormal epigenetic regulation of specific genes, such as *IGFBP3*, due to its chronic effects. We can also speculate that cells with suppressed *IGFBP3* expression due to Aβ-induced epigenetic modifications could be vulnerable to Aβ toxicity, and chronic exposure to Aβ protein could lead to damage or death in AD-related brain regions.

In this study, we demonstrate for the first time in AD an anti-apoptotic role of IGFBP3 that protects brain cells from toxic Aβ_1**–**42_ as well as *IGFBP3* modification by DNA methylation-dependent regulation. These findings suggest that Aβ-induced epigenetic alteration of *IGFBP3* is a plausible mechanism contributing to the pathogenesis of AD.

## Supporting Information

Figure S1 **Native PAGE analysis of the aggregation states of Aβ_1–42_ peptides freshly dissolved (A) or incubated at 4°C for 24 h (B).**
(TIF)

Figure S2 **The expression of beta-amyloid (Aβ) in H4 and H4-sw cells.** The secreted Aβ_1**–**40_ and Aβ_1**–**42_ peptides were determined by ELISA. Data are the means ± standard deviation of triplicate experiments.
(TIF)

Figure S3 **Endogenous Igfbp3 expression has not been altered by treatment of oligomeric Aβ_1–42_ for 24h.** Rat hippocampal neuronal primary cells in supplements-free media were treated for 24 h with or without 500 nM oligomeric Aβ_1**–**42_. After treatment with or without Aβ_1**–**42_, *Igfbp3* mRNA expression was measured using qPCR (A). Igfbp3 protein expression was detected using western blot analyses. Representative results are illustrated and values from densitometric analyses after normalization to β-actin are reported relative to that of untreated controls (B). Data are shown as the mean ± SD (n = 3).
(TIF)

Supporting Information S1 Supplementary Materials and Methods (DOCX)

References S1 (DOCX)
