# Supplementary figures and images for: Amyloid Beta-Mediated Epigenetic Alteration of Insulin-Like Growth Factor Binding Protein 3 Controls Cell Survival in Alzheimer's Disease
Source: PLoS One. 2014 Jun 25;9(6):e99047. doi: 10.1371/journal.pone.0099047 (PMC4070895; doi:10.1371/journal.pone.0099047)

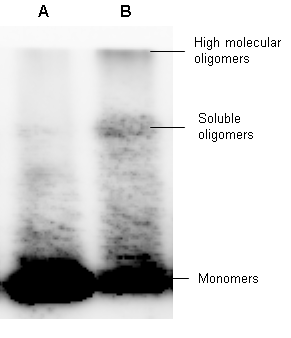

Supplement: Figure S1 — Native PAGE analysis of the aggregation states of Aβ1–42 peptides freshly dissolved (A) or incubated at 4°C for 24 h (B). (TIF) [file pone.0099047.s001.tif]

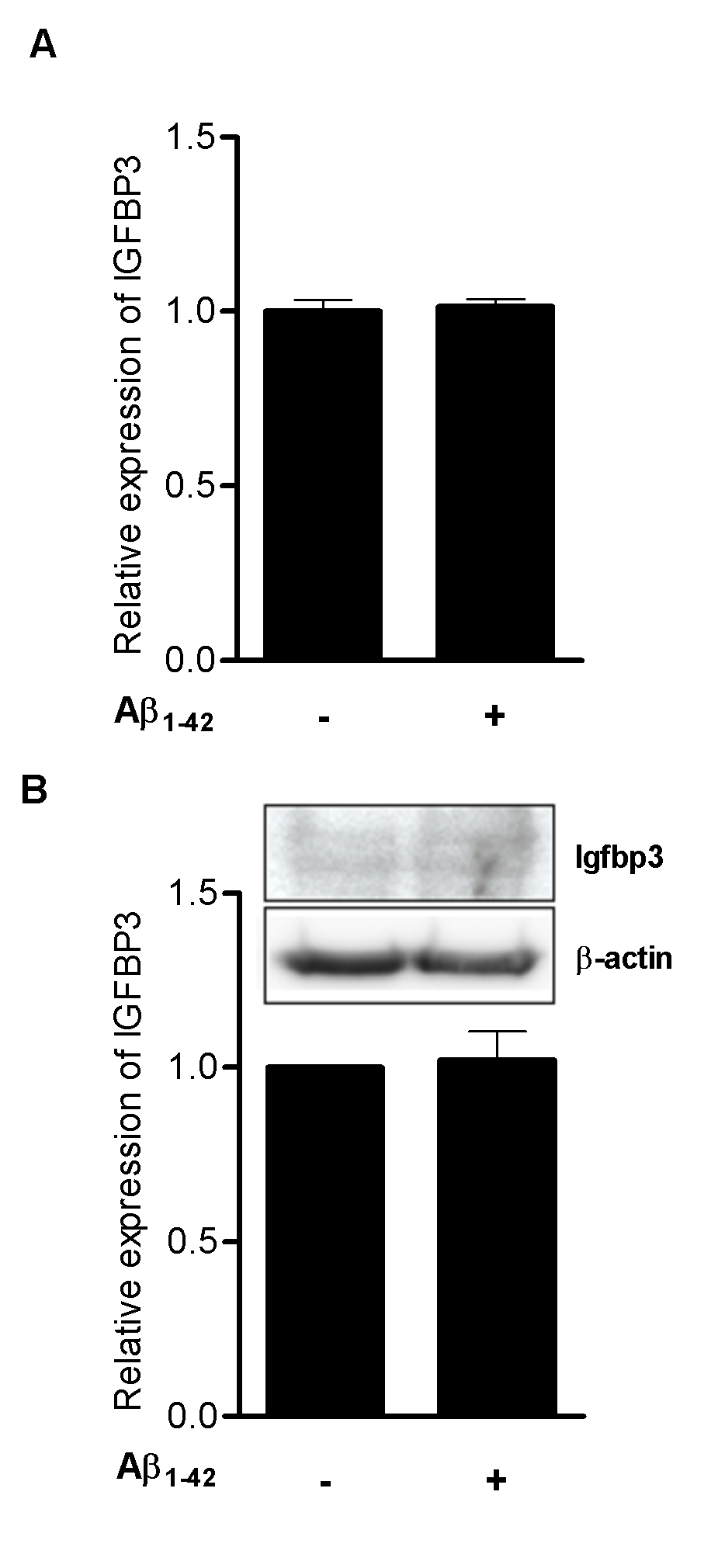

Supplement: Figure S3 — Endogenous Igfbp3 expression has not been altered by treatment of oligomeric Aβ1–42 for 24h. Rat hippocampal neuronal primary cells in supplements-free media were treated for 24 h with or without 500 nM oligomeric Aβ1–42. After treatment with or without Aβ1–42, Igfbp3 mRNA expression was measured using qPCR (A). Igfbp3 protein expression was detected using western blot analyses. Representative results are illustrated and values from densitometric analyses after normalization to β-actin are reported relative to that of untreated controls (B). Data are shown as the mean ± SD (n = 3). (TIF) [file pone.0099047.s003.tif]
