# Supplementary material for: Amyloid Beta-Mediated Epigenetic Alteration of Insulin-Like Growth Factor Binding Protein 3 Controls Cell Survival in Alzheimer's Disease
Source: PLoS One. 2014 Jun 25;9(6):e99047. doi: 10.1371/journal.pone.0099047 (PMC4070895; doi:10.1371/journal.pone.0099047)
Supplement: Supporting Information S1 Supplementary Materials and Methods — (DOCX) [file pone.0099047.s004.docx]

**Supporting information**

**Materials and Methods**

**Native PAGE and western blot analysis of beta-amyloid**

Aβ_1-42_ peptides (200 ng) freshly dissolved or incubated 4 °C for 24 h were separated by native polyacrylamide gel electrophoresis (PAGE) using 5% stacking and15% running gels with a buffer consisting of 0.025M Tris and 0.192 M glycine (pH 8.3) at 100 V for 3.5 h and transferred to polyvinylidene fluoride (PVDF) membranes. Membranes were blocked in 5% skim milk in Tris-buffered saline with 0.1% Tween 20 (TBST) and subsequently incubated overnight at 4°C with the amyloid beta monoclonal antibody (4G8 1:2000, Covance #SIG-39200). After washing, the membranes were incubated with anti-mouse secondary antibody conjugated to horseradish peroxidase for 1 h at room temperature. Chemiluminescence was detected using Super Signal West Dura substrate (Thermo Scientific) according to the manufacturer's protocol. Bands were visualized using a Luminescent Image analyser LAS-300 (General Electric) and quantified using Image Gauge software (Science Lab).

**Beta- amyloid (Aβ) quantitation (enzyme-linked immunosorbent assay)**

Aβ_1-40_ and Aβ_1-42_ in the culture media were quantitated as described [1] using an enzyme-linked immunosorbent assay kit (Biosource International) according to the manufacturer’s instructions.

**Western blot analysis of rat Igfbp3**

Proteins (40–50 μg) were resolved using denaturing 10% sodium dodecyl sulfate- polyacrylamide gel electrophoresis (SDS-PAGE) and transferred to polyvinylidene fluoride (PVDF) membranes. Membranes were blocked in 5% skim milk in Tris-buffered saline with 0.1% Tween 20 (TBST) and subsequently incubated overnight at 4°C with the following primary antibodies: goat anti-mouse Igfbp3 polyclonal antibody (1:1000, R&D Systems #AF775) and mouse anti-β-actin monoclonal antibody (1:2000, Sigma-Aldrich #A2066). After washing, the membranes were incubated with secondary antibodies conjugated to horseradish peroxidase for 1 h at room temperature. Chemiluminescence was detected using Super Signal West Dura substrate (Thermo Scientific) according to the manufacturer's protocol. Bands were visualized using a Luminescent Image analyser LAS-300 (General Electric) and quantified using Image Gauge software (Science Lab).
