# Supplementary material for: Amyloid Beta-Mediated Epigenetic Alteration of Insulin-Like Growth Factor Binding Protein 3 Controls Cell Survival in Alzheimer's Disease
Source: PLoS One. 2014 Jun 25;9(6):e99047. doi: 10.1371/journal.pone.0099047 (PMC4070895; doi:10.1371/journal.pone.0099047)
Supplement: References S1 — (DOCX) [file pone.0099047.s005.docx]

**References**

1. Kim M-J, Chae SS, Koh YH, Lee SK, Jo SA (2010) Glutamate carboxypeptidase II: an amyloid peptide-degrading enzyme with physiological function in the brain. The FASEB Journal 24: 4491-4502.
